# CPEB3-mediated MTDH mRNA translational suppression restrains hepatocellular carcinoma progression

**DOI:** 10.1038/s41419-020-02984-y

**Published:** 2020-09-23

**Authors:** He Zhang, Chendan Zou, Zini Qiu, Fang E, Qiang Li, Miao Chen, Dayong Wang, Qinrui Tan, Wanli Yin, Cedric Matunda, Hefei Wang, Yongjian Zhang, Chao Zhan, Chuxuan Wang, Yue Wu, Xiuchen Xuan, Yayan Wang, Chaoxia Zou, Guixiang Lv, Xu Gao

**Affiliations:** 1 Department of Biochemistry and Molecular Biology, Harbin Medical University, Harbin, Heilongjiang 150081 China; 2 Department of General Surgery, The Second Affiliated Hospital of Harbin Medical University, Harbin, Heilongjiang 150081 China; 3 Intensive Care Unit, Affiliated Hospital of Zunyi Medical University, Guizhou, Zunyi 563000 China; 4 Department of Biochemistry and Biotechnology, Technical University of Kenya, P.O. Box 52428-00200, Nairobi, Kenya; 5 Department of Hepatobiliary and Pancreas, Tumor Hospital of Harbin Medical University, Harbin, Heilongjiang 150081 China; 6 Translational Medicine Research and Cooperation Center of Northern China, Heilongjiang Academy of Medicine Sciences, Harbin, Heilongjiang 150081 China; 7 Key Laboratory of Cardiovascular Medicine Research of Harbin Medical University, Ministry of Education, Harbin, Heilongjiang 150081 China; 8 Key Laboratory of Preservation of Human Genetic Resources and Disease Control in China, Harbin Medical University, Ministry of Education, Harbin, Heilongjiang 150081 China

**Keywords:** Tumour biomarkers, Tumour-suppressor proteins

## Abstract

Cytoplasmic polyadenylation element-binding protein 3 (CPEB3) is a sequence-specific RNA-binding protein. We had reported that CPEB3 is involved in hepatocellular carcinoma (HCC) progression. However, the underlying mechanisms of CPEB3 in HCC remain unclear. In this study, we firstly performed RNA immunoprecipitation to uncover the transcriptome-wide CPEB3-bound mRNAs (CPEB3 binder) in HCC. Bioinformatic analysis indicates that CPEB3 binders are closely related to cancer progression, especially HCC metastasis. Further studies confirmed that metadherin (MTDH) is a direct target of CPEB3. CPEB3 can suppress the translation of MTDH mRNA in vivo and in vitro. Besides, luciferase assay demonstrated that CPEB3 interacted with 3′-untranslated region of MTDH mRNA and inhibited its translation. Subsequently, CPEB3 inhibited the epithelial–mesenchymal transition and metastasis of HCC cells through post-transcriptional regulation of MTDH. In addition, *cpeb3* knockout mice are more susceptible to carcinogen-induced hepatocarcinogenesis and subsequent lung metastasis. Our results also indicated that CPEB3 was a good prognosis marker, which is downregulated in HCC tissue. In conclusion, our results demonstrated that CPEB3 played an important role in HCC progression and targeting CPEB3-mediated mRNA translation might be a favorable therapeutic approach.

## Introduction

A growing body of evidence suggests that tumor cells can highjack post-transcriptional machinery to trigger reprogramming of gene expression when challenged by internal or external stimuli, which in turn enhances tumor cell proliferation and metastasis ultimately^1,2^. The CPEB family of proteins (CPEB1–4) are all sequence-specific RNA-binding proteins. CPEB1 is the first one identified as critical roles in cell cycle control and oocyte maturation^3^. CPEB3 and CPEB4 have also been recognized to play an important role in neuron cells^4^. Specifically binding to cytoplasmic polyadenylation elements (CPEs), CPEBs can further recruit several functional proteins to regulate the poly(A) tail length of target mRNAs, resulting in translational activation or repression of its targets^5,6^. The C terminus, which includes two RNA recognition motif and a zinc-finger domain, is highly conserved among CPEBs^7^. While the highly variable N terminus containing regulatory sites may contribute to the distinct functions of CPEB family members^4^. Despite the structural similarities among CPEBs, the expression of CPEBs in different malignancies is highly variable as well as the functions of CPEBs^8,9^. Evidence has shown that CPEB1 is likely to act as a tumor suppressor, while CPEB2 and CPEB4 seem to be oncogenes^7^.

CPEB3-controlled translation regulates synaptic plasticity and memory formation^10^. However, whether the CPEB3-controlled post-transcriptional machinery implicated in cancer progression is poorly explored. A meta-analysis that integrated the data from 42 microarrays showed that CPEB3 expression is ubiquitously downregulated in cancers related to digestive system^11^. In our previous work, we uncovered that CPEB3 is a novel target of miR-107 and inhibits hepatocellular carcinoma (HCC) progression^12^. Tang et al.^13^ also have demonstrated that CPEB3 overexpression suppresses the proliferation and migration of HCC cells. However, the underlying mechanism of CPEB3 involved in HCC suppression remains to be elucidated.

Metadherin (MTDH) is regarded as a key factor implicated in promoting metastasis in various malignancies, including breast cancers, esophageal cancers, gliomas, bladder cancers, and HCC^14,15^. Compared with normal liver, overexpressed MTDH is found in HCC, which is correlated with patients’ poor prognosis and recurrence^16,17^. Besides, accumulating evidence suggests that MTDH is tightly involved in epithelial–mesenchymal transition (EMT)^18^. More importantly, MTDH is the direct target of CPEB1 in the rat glioblastoma cell line CNS1^19^, whose 3′-untranslated region (3′-UTR) contains an abundance of canonical and noncanonical CPE elements. Here, we detected the transcriptome-wide targets of CPEB3 in HCC cells. Among these potential targets, MTDH was firstly concerned because of its special functions in tumor progression and its sequence feature of mRNA 3′-UTR. Our results indicated that CPEB3 can specifically target the 3′-UTR of MTDH mRNA to suppress its protein expression resulting in a suppressed metastatic ability of HCC cells. Then, CPEB3-controlled post-transcriptional machinery may provide new insights into developing new strategies for treating metastatic HCC.

## Materials and methods

### Patients and clinical samples

Thirty human HCCs and the paired non-tumorous samples were collected from Heilongjiang Cancer Hospital (Harbin, China). The consent from each patient and approval from the local ethics committee was obtained. The characteristics of all patients are shown in Supplementary Table S1 in detail.

### Cell culture and reagents

HepG2, Hep3B, and HEK293T cell lines were purchased from American Type Culture Collection (Manassas, VA, USA). Huh7 cell line was purchased from China Center for Type Culture Collection (Shanghai, China). All cells were maintained in Dulbecco’s modified Eagle’s medium (DMEM, Gibco, Carlsbad, CA, USA). The medium was supplemented with 10% fetal bovine serum (FBS, Gibco), 100 U/mL penicillin, and 100 μg/mL streptomycin. All cells were maintained in a humidified incubator containing 5% CO_2_ at 37 °C. Cell lines used in this study were regularly authenticated by morphological observation and tested for the absence of mycoplasma contamination.

### Plasmid construction, oligonucleotide synthesis, and transfection

Full-length human CPEB3 mRNA (NM014912) coding sequence followed by 1 × 3′-FLAG tag was synthesized and cloned into pcDNA3.1 vector to construct the recombinant pcDNA3.1-CPEB3-FLAG by Genechem (Shanghai, China). Lentiviral vector pLVX-CPEB3-FLAG was generated by subcloning CPEB3-FLAG fragments into pLVX-DsRed vector at restriction sites *Hin*dIII/*Xho*I. pcDNA3.1(+)-MTDH was established by amplification of human MTDH mRNA CDS region using primer MTDH-Clone-F/R and cloned into pcDNA3.1(+) backbone at restriction sites *Eco*RI/*Xba*I. The oligonucleotides used in the construction of CPEB3 knockdown vector (pLKO.1-shNC/CPEB3#1/CPEB3#2/CPEB3#3) were shown in Supplementary Table S2. Transient transfection was achieved by Lipofectamine^TM^ 3000 (Thermo Fisher Scientific) reagent as the manufacturer’s instructions.

### Lentivirus production and stable cell lines generation

Lentivirus was packaged according to a standard packaging system. psPAX2 7.5 μg, pMD2.G 2 μg, and lentiviral vector of 10 μg (pLVX-DsRed, pLVX-CPEB3-FLAG, or pLKO.1-shNC/CPEB3#1/CPEB3#2/CPEB3#3) were transfected into HEK293T cells with 30 μL of Lipofectamine^TM^ 2000 in 10 cm dish. The virus was collected and filtered 24 and 48 h after transfection.

For stable cell line establishment, cells were infected with the medium composed of half virus-containing medium and half fresh medium with 5 μg/mL polybrene. Puromycin was added 72 h after infection at a final concentration of 5 μg/mL to screen the positively infected cells for 7 days.

### Generation of *cpeb3* knockout mouse by CRISPR/Cas9

The sgRNA- (sequence: ATCCCATCTGCATCTTGTCG) specific targeting the coding region of the mouse *cpeb3* gene was designed. The sense and antisense oligos were then synthesized (Sangon Biotech, Shanghai, China) and annealed in NEB buffer 2 (Beverly, MA, USA). The annealed sgRNA oligos were subsequently ligated into pX330 vector at the restriction site *Bbs*I. The vectors containing sgRNA were verified by DNA-sequencing. The vectors with Cas9 mRNA and sgRNAs were microinjected into fertilized embryos of C57BL/6J mice. All mice were genotyped 2 weeks after birth. *cpeb3* gene editing was verified by T7 endonuclease I (T7E1) assay^20^, Sanger sequencing, and Western blotting. The gRNA targeting region was amplified by primers (F: TAGTGCCTGTGGACCAGGTG; R: CCAGCCCGACTTGTTCTTCT). Then, it was mixed with the PCR product of the pre-genotyping mice with the PCR product from wild-type mice (*cpeb3*^+/+^) to improve the cleavage of T7EI during the assay. Mice were kept in a well-controlled specific-pathogen-free facility with 40–60% humidity, 37 °C constant temperature, and a 12 h light/dark cycle. The animal experiments were approved by the local ethics committee of Harbin Medical University.

### Chemically induced HCC model

The methodology of the chemically induced HCC model was well adopted by our group and described previously^21^. In brief, the mice with different genotypes (*cpeb3*^+/+^, *cpeb3*^+/^^−^, and *cpeb3*^−/−^) received a single injection of diethylnitrosamine (DEN) at 25 mg/kg 13 days after birth. After the injection, the mice were cultured under continuous observation for an additional 36 weeks and then sacrificed. The livers and lungs were then dissected, photographed, and fixed.

### Quantitative real-time PCR analysis

Detailed procedures for quantitative real-time PCR analysis (qRT-PCR) were carried out as previously described^12^. All samples were performed in triplicates and mRNA expressions were analyzed using 2^−ΔΔCT^ calculation method after normalized with glyceraldehyde-3-phosphate dehydrogenase or actin house-keeping gene expressions. The primers used in qRT-PCR are shown in Supplementary Table S3.

### Western blot analysis

The details for conducting Western blot as previously described^12^. Briefly, 30–80 μg protein was loaded in polyacrylamide gel (10–12.5% conc.), followed by electrophoresis and transfer. The primary and horseradish peroxidase (HRP)-linked secondary antibodies used in this study are described in Supplementary Table S4.

### Colony formation assay

For colony formation assay, cells were seeded in 6-well plates (Corning Life Sciences, USA) at a density of 1000 cells/dish and incubated for 10–14 days with continuous observation. The colonies were then fixed in methanol, stained with 0.1% crystal violet (Sigma, St. Louis, MO, USA), and counted by Image J.

### Immunofluorescence and immunohistochemistry

For immunofluorescence (IF), cells were fixed with 4% paraformaldehyde for 30 min at room temperature and then permeabilized by 0.1% Triton X-100. The cells then incubated with primary antibodies (rabbit anti-E-cadherin, CST, #3195) overnight at 4 °C in a humidified box. After incubation, cells were incubated with fluorescence-labeled secondary antibodies for 1 h at room temperature in dark. The nuclei were stained by 4′,6-diamidino-2-phenylindole (DAPI) and the cells were visualized by confocal microscopy.

Immunohistochemistry (IHC) was performed following the standard method. Briefly, mouse liver tissues were fixed in 4% paraformaldehyde and then embedded in paraffin. The 5-μm tissue sections were made and stained using anti-MTDH antibodies (Abcam, ab45338) and HRP-linked secondary antibodies. The tissue sections were also stained using hematoxylin and eosin. The stained slides were mounted using neutral balsam (Solarbio, G8590) and imaged by an inverted microscope.

### Migration and invasion assay

Transwell chamber (8 μm pore size, Corning Incorporated, Corning NY, USA) was used. For migration assay, cells (2.0 × 10^4^, 1.0 × 10^5^, and 1.0 × 10^4^ for Huh7, HepG2, and Hep3B, respectively) were suspended in 200 μL of FBS free medium, and then the cells were subsequently pipetted into the upper chamber. Besides, for eliminating the effect of cell proliferation, Huh7 and HepG2 cells were also pre-treated with medium containing 10 μg/mL of mitomycin c (S8146, Selleck Chemicals, Houston, TX, USA) for 2 h to block cell proliferation, and then subjected to transwell migration assay.

For invasion assay, membranes were coated with a mixture containing Matrigel and FBS free DMEM medium at 1:4 ratios. Then, 1.0 × 10^5^ of Huh7 and 5.0 × 10^4^ Hep3B cells were suspended in 200 μL of FBS free medium and pipetted into the upper chamber. Cells were incubated for 36 h (HepG2) or 24 h (Huh7 and Hep3B) and then fixed, stained, and counted as previously described^12^.

### RNA immunoprecipitation and sequencing

RNA immunoprecipitation (RIP) was performed using Magna RIP™ RNA-Binding Protein Immunoprecipitation Kit (Merck Millipore, Billerica, MA, USA) as per the manufacturer’s instructions. In brief, Huh7 cells were transfected with pcDNA3.1(+)-CPEB3-FLAG and incubated for 72 h, and then lysed by RIP lysis buffer. CPEB3-associated mRNAs were immunoprecipitated with mouse anti-FLAG antibodies (A00187, GenScript, Piscataway, NJ, USA). The mouse immunoglobulin G (IgG) was served as a control to monitor the unspecific interaction. Then, the quality of precipitated RNA was determined by NanoDrop 2000c and the RIP effectiveness was confirmed by Western blot. The immunoprecipitated RNA was sequenced by Illumina Hiseq 2500 in single-end 50 bp and ~90 M RAW reads were obtained (NovoGene, Beijing, China).

### Luciferase activity assay

For the construction of luciferase reporter vector, RNA was extracted from HEK293T cells and reverse transcribed to cDNA. Two pairs of primers (MTDH-UTR1-F/R, MTDH-UTR2-F/R) were designed to amplify the distal and proximal region of MTDH mRNA 3′-UTR. Besides, two pairs of primers (MTDH-RUTR1F/R, MTDH-RUTR2F/R) were designed to achieve a reverse complement insertion of the cloned fragments. Then, the 3′-UTR fragments and its corresponding reverse complement fragments (as controls) were cloned into pmir-GLO luciferase reporter vector (Promega, Madison, WI, USA) at restriction sites *Xba*I and *Sac*I. The insertion sequences were further verified by sequencing. Primers used in the construction of the reporter vector are shown in Supplementary Table S3.

For luciferase activity determination, pcDNA3.1(+)-CPEB3-FLAG or pcDNA3.1(+) was co-transfected with luciferase reporter vectors containing MTDH mRNA 3′-UTR fragment or control sequence in HEK293T cells. The transfected cells were incubated for 48 h and luciferase activity was determined by the Dual-Luciferase Reporter Assay System (Promega) following the manufacturer’s instructions.

### Bioinformatics

The Cancer Genome Atlas (TCGA) expression data (fragments per kilobase of exon model per million reads mapped) and corresponding clinical information of each tumor type were downloaded from TCGA Data Portal (https://cancergenome.nih.gov/) and transformed to TPM (Transcripts Per Millions) value. The expression profile GSE14520, GSE9843, GSE20017^22–24^, and the corresponding clinical information were downloaded from Gene Expression Omnibus by R package GEOqurey^25^. The data exclusion criteria for TCGA and GEO data analysis were described in Supplementary Materials and methods.

Differential expression was analyzed by extraction of the expression value from those datasets and statistical power was determined by paired or non-paired Student’s *t* test. For global CPEB expression analysis, the expression value of CPEBs was extracted from TCGA. Datasets without expression value of normal specimens were excluded from the study. The mean expression value of CPEBs in tumor and normal sample of each tumor type was calculated. The differential expression was presented as the log 2 ratio of the mean expression value of CPEBs in tumor specimens and normal specimens. For survival analysis, patients in TCGA-LIHC and GSE14520 were grouped based on the mRNA expression level of CPEB3 and MTDH among all patients (low quartile, low expression group; upper quartile, high expression group). The Kaplan–Meier plots were plotted by GraphPad Prism 6.0, and the log-rank test was used to examine the statistical significance. The survival map of CPEB family of protein was plotted using GEPIA^26^ online tools (http://gepia.cancer-pku.cn/).

For RIP-sequencing (RIP-Seq) data analysis, clean reads were obtained from NovoGene and mapped to hg38 reference genome by using TopHat2^27^, and transcripts were quantified by HtSeq^27^ with the default settings. The details for data preprocessing are as described in Supplementary Table S5. The differential gene expression was analyzed by DeSeq2^28^. Transcripts with log 2 (IP/Input) > 0 and false discovery rate (FDR) < 0.01 were considered to interacting with CPEB3 and subjected to downstream analysis. For CPE elements analysis, the curated 3′-UTR sequences of human mRNAs were downloaded from UCSC Table Browser (http://genome.ucsc.edu). The CPEC (sequence: UUUUA_1–2_U), CPENC (sequences: UUUUAAAU/UUUUACU/UUUUCAU), Hex (sequence: AAUAAA/AUUAAA), and PBE (sequence: UGUAAAUA/UGUAUAUA) in 3′-UTRs of CPEB3 binders or CPEB3 non-binders were quantified by package biostring in R, and the Fisher’s exact test was performed to exam the statistical significance. The visualization of RIP-Seq result was achieved by converting and normalizing alleged BAM files to bigWig files by using Deeptools, and then the bigWig files were visualized by Integrative Genomics Viewer.

For gene set enrichment analysis (GSEA), gene symbols of CPEB3 binders were subjected to clusterProfiler^29^ in R. The pre-ranked gene list was generated by Limma output using “log FC” as the ranking metric. For gene signature survival analysis, the CPEB3 binders (656 genes) were analyzed by GEPIA online tools^26^, the patients were grouped based on the expression of the gene signature, and the overall and disease-free survival time was analyzed. For over-representation analysis (ORA), gene symbols of 656 identified CPEB3 targets were subjected to WebGsetalt database^30^. The enrichment of KEGG pathway. Reactome pathway and GO celluar components were analyzed. For principal component analysis, the expression data of the CPEB3 binders were processed using pca3d package in R.

### Statistical analysis

All data were presented as mean ± SEM and performed in triplicates at least. Differences between two groups were compared by two-tailed Student’s *t* test or Wilcoxon test. For multiple group comparison, one-way analysis of variance was performed. Statistical analysis was accomplished by GraphPad Prism 6.0 and R 3.5.1. *P* value less than 0.05 was considered as statistically significant.

## Results

### The characterization of transcriptome-wide CPEB3-bound mRNAs

**Fig. 1:**
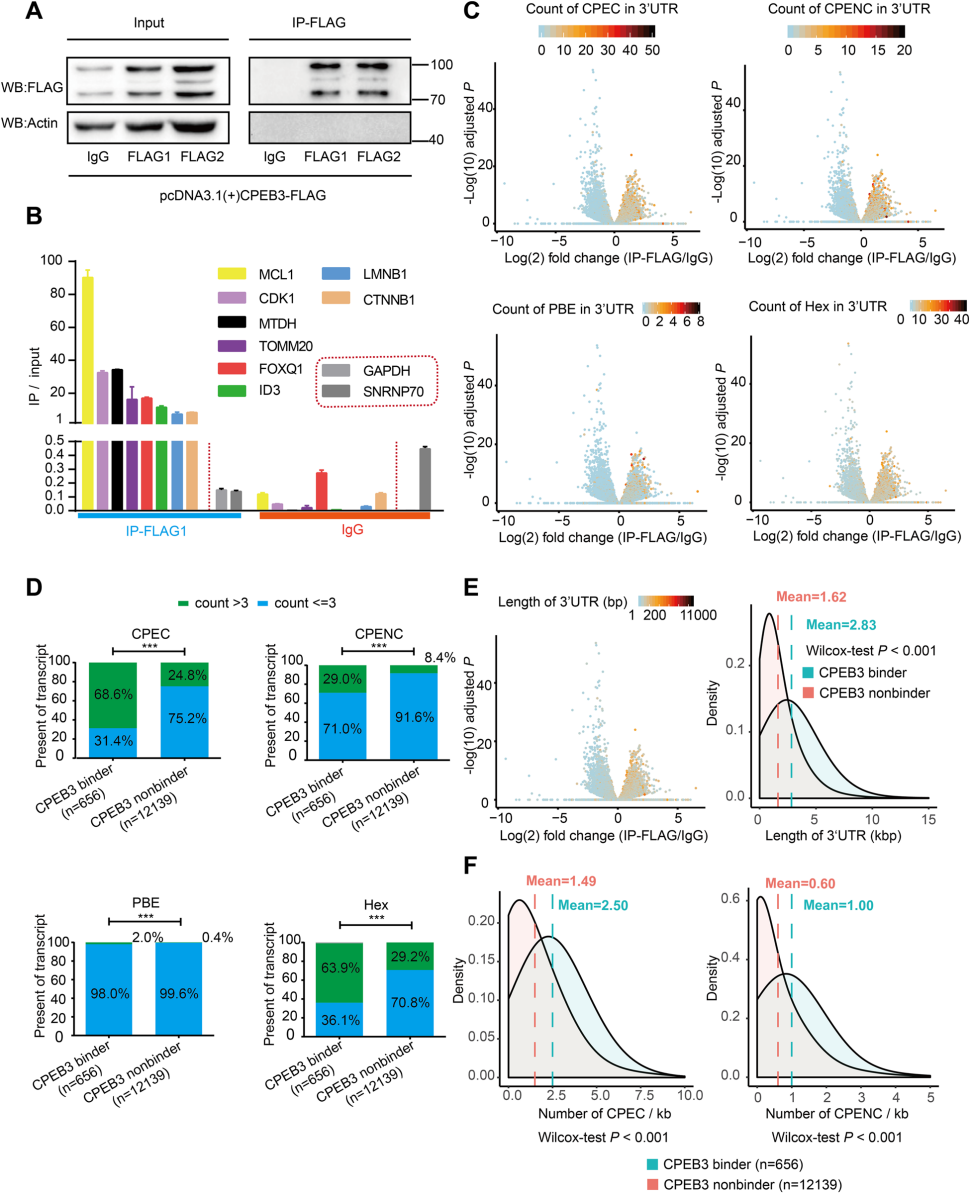
Transcriptome-wide characterization of CPEB3-bound mRNAs. **a** Verification of RNA immunoprecipitation for IP samples (IP-FLAG1 and IP-FLAG2) and the control (IgG) by Western blot analysis. **b** Validation by qRT-PCR of mRNAs bound to CPEB3. The fold enrichment of target sequences in the CPEB3 immunoprecipitants compared to IgG and to the input fraction is shown. GAPDH and SNRNP70 are negative controls. **c** Volcano plots showing the relative enrichment of mRNAs in IP-FLAG and IgG samples according to the count of CPEC, CPENC, PBE, and Hex in the 3′-UTR of mRNA. **d** Bar plots showing the counts (>3 or ≤3) of CPEC, CPENC, PBE, and Hex elements in the 3′-UTR of the CPEB3 binders and non-binders. (***P < 0.001, Fisher exact test). **e** Left panel, volcano plots showing the relative enrichment of mRNAs in IP-FLAG and IgG, the length of 3′-UTR of mRNA are indicated by colored dots. Right panel, density plot showing the length of 3′-UTR of mRNA in CPEB3 binder and non-binder (Wilcoxon test *P* < 0.001). **f** Density plot showing the abundance of CPEC and CPENC (presented as per kilobase) in CPEB3 binder and non-binder (Wilcoxon test *P* < 0.001). CPEC consensus cytoplasmic binding element, CPENC non-consensus cytoplasmic bind element, Hex hexanucleotide AAUAAA, PBE pumilio-binding element, MCL1 myeloid cell leukemia sequence 1, CDK1 cyclin-dependent kinase 1, MTDH metadherin, TOMM20 translocase of outer mitochondrial membrane 20, FOXQ1 forkhead box Q1, ID3 inhibitor of DNA binding, LMNB1 lamin B1, CTNNB1 catenin beta 1, GAPDH glyceraldehyde-3-phosphate dehydrogenase, SNRNP70 small nuclear ribonucleoprotein U1 subunit 70.

To obtain a transcriptome-wide picture of potential CPEB3 targets in HCC, we precipitated CPEB3 mRNA complex from Huh7 cell extracts after transiently transfected with pcDNA3.1-CPEB3-FLAG and detected the associated mRNAs by Illumina Hiseq 2500 sequencing (Fig. 1a). We identified that 656 mRNAs were significantly (*P* < 0.05) associated with CPEB3 (referred to as CPEB3 binders) when compared with IgG control group (Supplementary Table S6). Then, we validated eight of the potential CPEB3 binders by qRT-PCR with a validation rate of 100% (Fig. 1b).

The well-known strategy that CPEBs bind to its target mRNAs is through high-specificity consensus CPE (CPEC) or non-consensus CPE (CPENC), which are frequently located in the 3′-UTR of mRNA^5^. In addition, the hexanucleotide AAUAAA (Hex) and additional *cis*-acting elements, such as the Pumilio-binding element (PBE) are also associated with CPEBs’ translational regulatory function^5^. Thus, we counted the number of CPEC, CPENC, Hex, and PBE elements in the 3′-UTR of both CPEB3 binders and CPEB3 non-binders. As expected, we found that the mRNAs enriched in the IP-FLAG samples had more CPEC, CPBNC, Hex, and PBE elements in their 3′-UTR than those enriched in the IgG samples (Fig. 1c). Next, we calculated that the proportion of these mRNAs containing more than three elements. The results showed that CPEC, CPENC, Hex, and PBE elements all involved in the interaction between CPEB3 and its binders. Interestingly, the count difference of CPEC and Hex between CPEB3 binder and non-binder was more obvious than CPENC and PBE (Fig. 1d). Besides, we also found the potential CPEB3 binders were more likely to have a relatively longer 3′-UTR than CPEB3 non-binders (Fig. 1e). The count of CPEC and CPENC elements per kilobase of the mRNA 3’-UTR in the CPEB3 binders was significantly more than that in the CPEB3 non-binders (Fig. 1f). The detailed results for the RIP-Seq analysis are shown in Supplementary Table S6. Based on the above results, we speculated that the length of the 3′-UTR and the abundance of CPEs could be important indicators to predict the probability of CPEB3 targets.

**Fig. 2:**
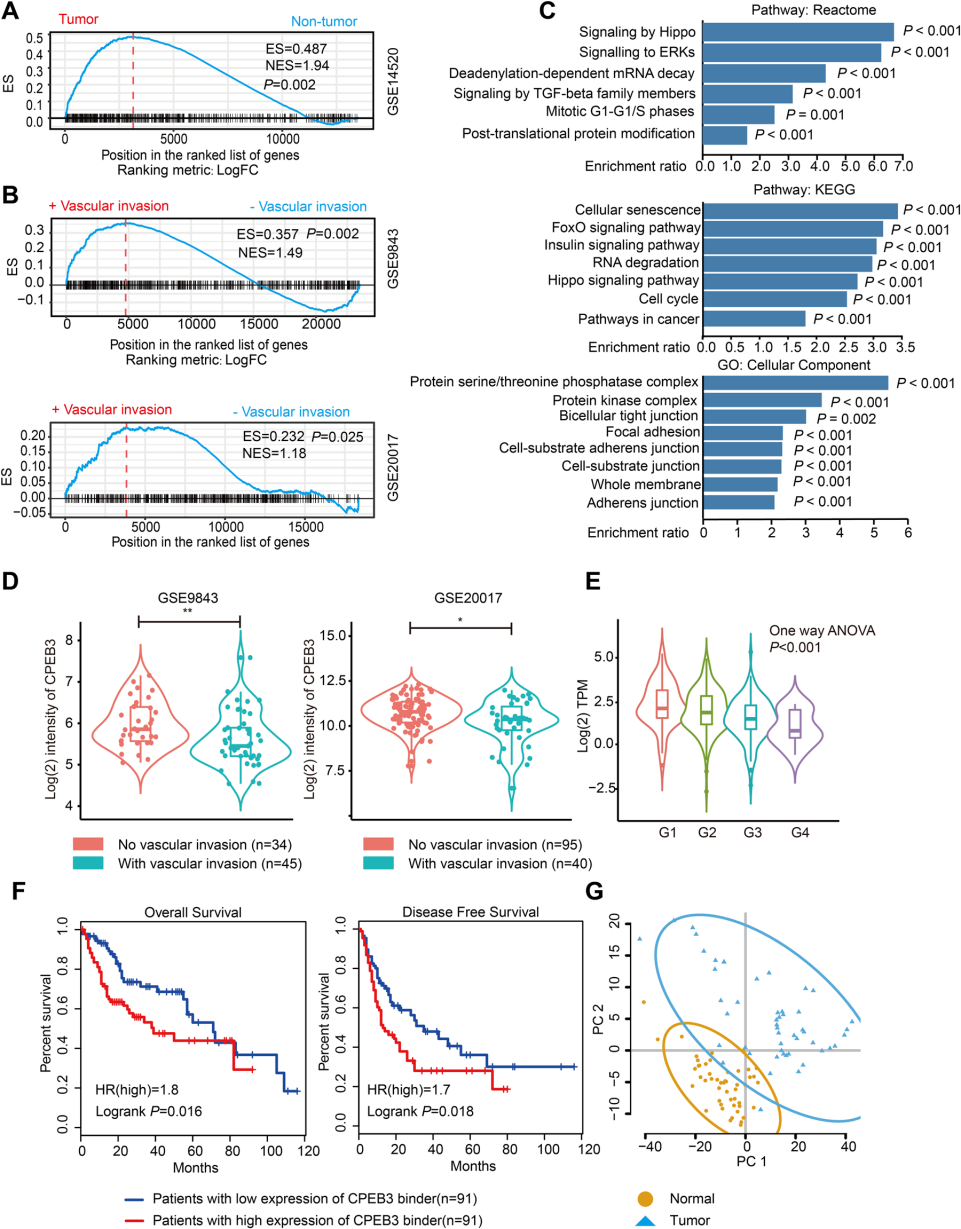
CPEB3-bound mRNAs are involved in HCC progression. **a** Gene set enrichment analysis (GSEA) showing the CPEB3 binders were enriched in tumor samples in GSE14520 dataset. **b** GSEA showing the CPEB3 binders were enriched in tumor samples with vascular invasion in GSE9843 and GSE20017 datasets. **c** Over-representation analysis (ORA) of CPEB3 binders against terms in Reactome pathway, KEGG pathway, and Gene Ontology cellular compartment. **d** Violin plots showing the CPEB3 expression in HCC with or without vascular invasion in GSE9843 and GSE20017 (independent Student’s *t* test). **e** Expression of CPEB3 in HCC samples with different histological grades in the TCGA-LIHC dataset (G1, *n* = 55; G2, *n* = 177; G3, *n* = 122; and G4, *n* = 12; *P* = 0.001, one-way ANOVA). **f** Survival analysis of HCC patients with high or low expression of CPEB3 binders by GEPIA online tools. **g** Principal component analysis for the expression profiles of 656 CPEB3 binders to distinguish HCC from normal samples in the TCGA-LIHC dataset (tumor = 50, normal = 50). *P < 0.05, **P < 0.01.

Next, we systemically analyzed the biological and clinical significance of CPEB3-bound mRNAs. A gene set defined by the CPEB3 binders was subjected to several bioinformatic analyses. GSEA analysis suggested that CPEB3 binders were enriched in tumor samples compared with normal samples in the GSE14520 dataset (Fig. 2a). More importantly, GSEA revealed that the CPEB3 binders were enriched in HCC tissues with vascular invasion in two independent datasets GSE9843 and GSE20017 (Fig. 2b). Furthermore, the ORA results indicated that CPEB3 binders were associated with many tumor-related signaling pathways (Fig. 2c). In addition, CPEB3 binders were enriched in bicellular junctions, focal adhesions, and cell-substrate adherens junctions (Fig. 2c), all of which are tightly related to cancer cell metastasis. The detailed results of the ORA analysis are shown in Supplementary Table S7.

We also found that CPEB3 was downregulated in HCC samples with vascular invasion in two independent datasets GSE9843 and GSE20017 (Fig. 2d). Meanwhile, decreased expression of CPEB3 mRNA was correlated with the rising grade of tumor malignancy in HCC (Fig. 2e). Also, survival analysis using online database GEPIA showed that patients with high expression of CPEB3-bound mRNAs (high expression of the 656 genes signature) had worse overall survival and disease-free survival time (Fig. 2f). Based on the expression of these 656 CPEB3-bound mRNAs, we could completely distinguish HCC samples from normal samples, which indicated that the mRNA expression of CPEB3 targeted mRNAs were different between HCC and normal tissue (Fig. 2g). Collectively, we inferred that numerous of onco-promoting mRNAs might shift to a translationally active state in HCC with low CPEB3 expression level, which in turns transited HCC cells from a low metastatic stage toward a more aggressive advanced stage. Thus, the dysregulation of CPEB3 will ultimately make tumor cells more aggressive.

### CPEB3 translationally suppresses MTDH protein expression in liver cells and HCC cells

**Fig. 3:**
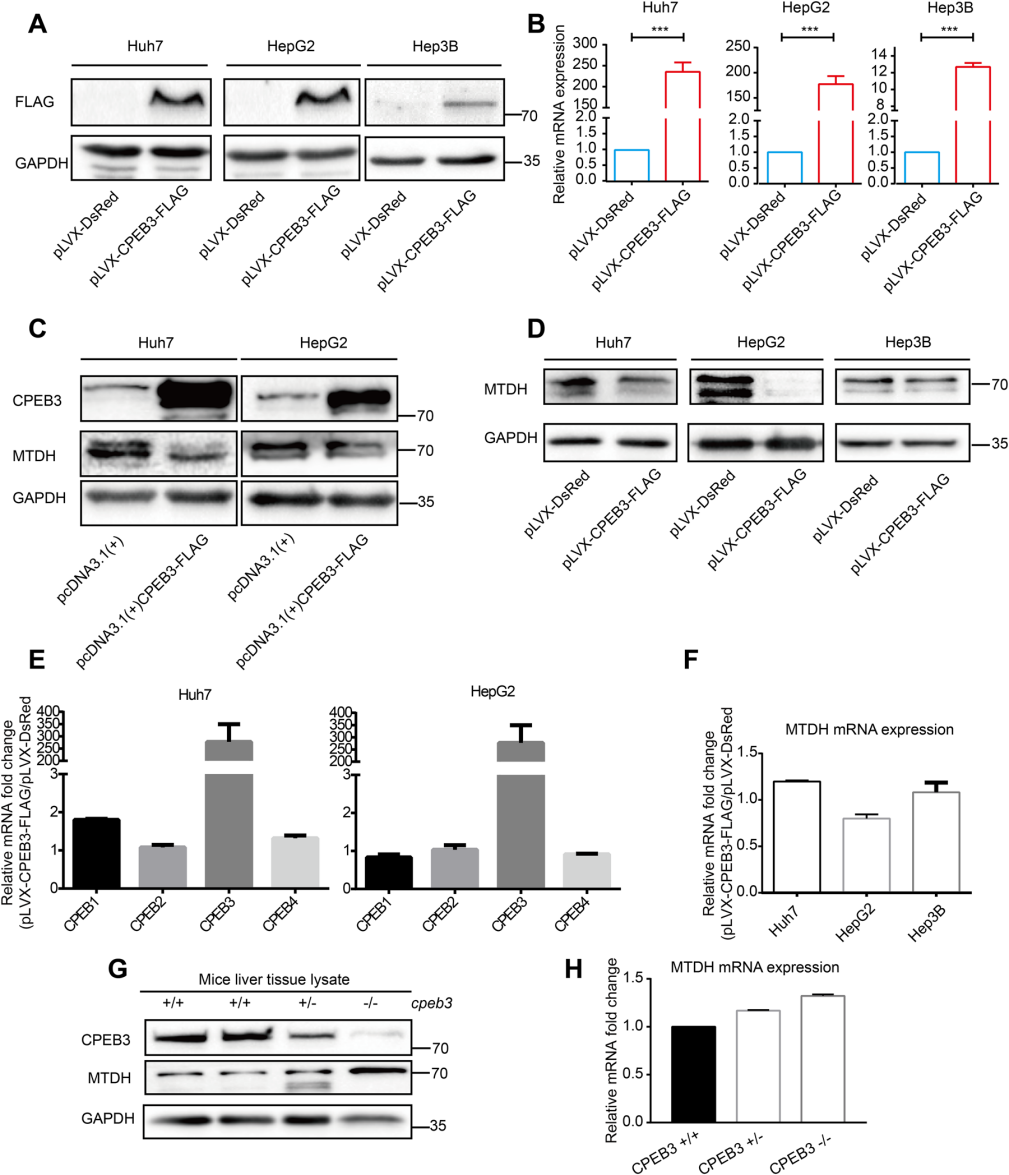
CPEB3 suppresses MTDH protein expression in normal liver tissues and HCC cells. **a**, **b** Verifications of stable CPEB3-overexpressed cell lines by Western blot and qRT-PCR (independent Student’s *t* test). **c** Western blot analysis of MTDH protein expression in transient CPEB3-overexpressed and control cell lines. **d** Western blot analysis of MTDH expression in Huh7, HepG2, and Hep3B cells with stably overexpressing of CPEB3. **e** The mRNA expression of CPEB1, CPEB2, CPEB3, and CPEB4 was analyzed by qRT-PCR in CPEB3-overexpressing Huh7 and HepG2 cell lines. **f** The mRNA expression of MTDH after CPEB3 overexpression was detected by qRT-PCR in Huh7, HepG2, and Hep3B cells. **g** Western blot analysis of the CPEB3 and MTDH expression in liver tissues from *cpeb3*^+/+^, *cpeb3*^+/−^, and *cpeb3*^−/−^ mice. **h** Analysis of MTDH mRNA expression in liver tissues from *cpeb3*^+/+^, *cpeb3*^+/−^, and *cpeb3*^−/−^ mice. For all figures, the results are summarized as the mean ± SEM of three independent experiments. ***P < 0.001.

To identify the direct targets of CPEB3, we next selected several predicted CPEB3 binders and assessed them by Western blot. The expression of CPEB3 in stably transfected HCC cell lines was verified by Western blot and qRT-PCR (Fig. 3a, b). We found whether transient (Fig. 3c) or stable (Fig. 3d) overexpression of CPEB3 dramatically inhibited the expression of MTDH protein in HCC cells. Meanwhile, no significant changes in CPEB1, CPEB2, and CPEB4 mRNA expression were observed after ectopically expressing CPEB3 in Huh7 and HepG2 cell lines, which may suggest that the expression of CPEBs is not interconnected (Fig. 3e). However, MTDH mRNA expression was also unchanged, (Fig. 3f). This result also supported the conclusion that CPEB3 regulates the translation of its targeted mRNA specifically at the post-transcriptional level without altering the mRNA expression.

To further validate the regulatory relationship between CPEB3 and MTDH, we detected the expression of CPEB3 and MTDH protein in *cpeb3* knockout (KO) mice. The construction and identification of CRISPR/Cas9-mediated CPEB3-null mice were shown in Supplementary Fig. S1. A sgRNA was designed to target the mouse *cpeb3* gene (Supplementary Fig. S1A). The genotype of *cpeb3* KO mice was determined by T7E1 assay and DNA-sequencing of the sgRNA targeting region (Supplementary Fig. S1B–D).

We found that the MTDH protein expression in *cpeb3*^−/−^ mice was increased compared with *cpeb3*^+/+^ and *cpeb3*^+/−^ mice (Fig. 3g). Consistent with the in vitro observations, the mRNA expression of MTDH has no significant difference between *cpeb3*^+/+^ and *cpeb3*^−/−^ mice (Fig. 3h).

### CPEB3 interacts with 3′-UTR of MTDH mRNA to inhibit its translation

**Fig. 4:**
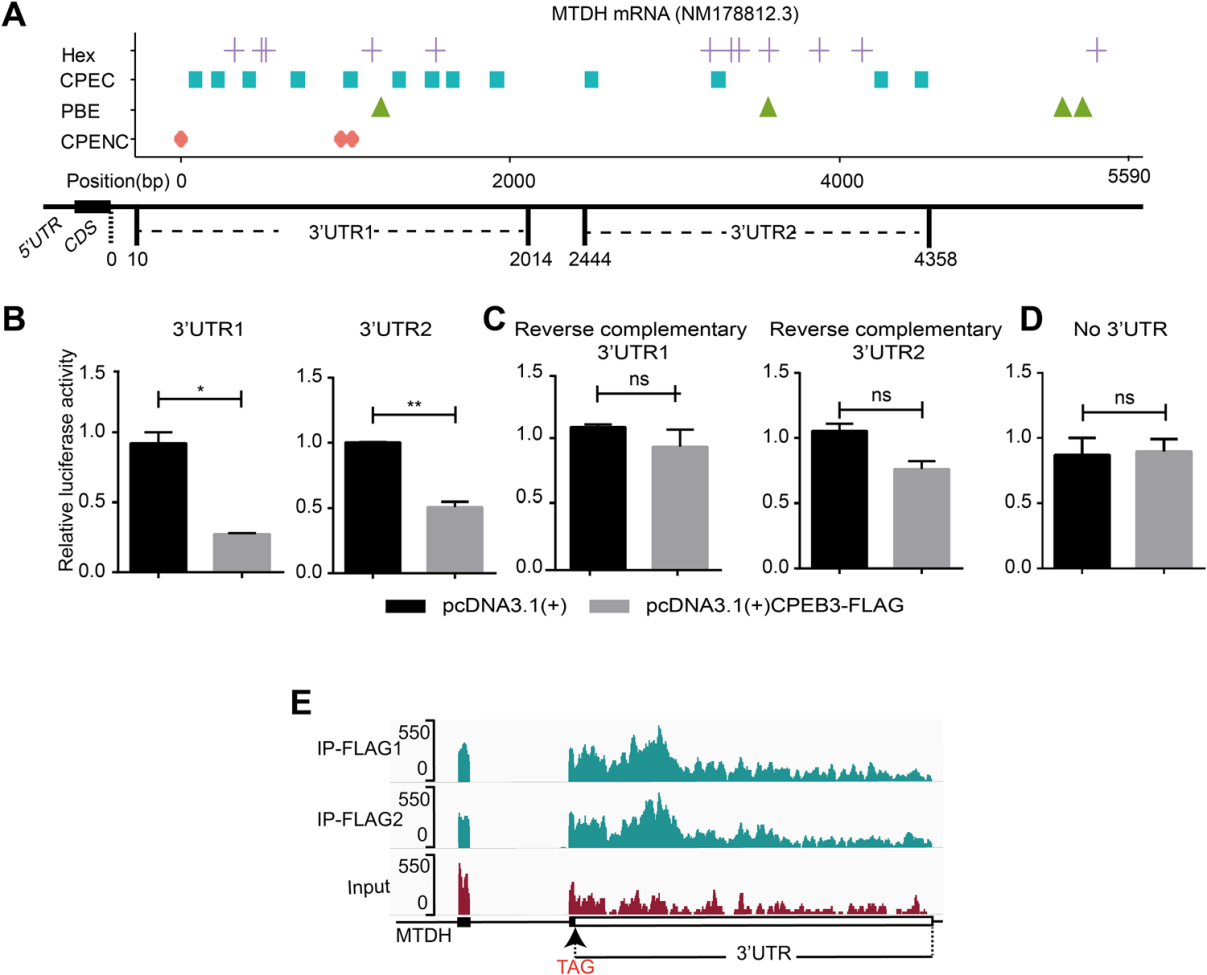
CPEB3 interacts with 3’UTR of MTDH mRNA to inhibit its translation. **a** Distribution of Hex, CPEC, PBE, and CPENC elements in the 3′-UTR of MTDH mRNA. The 3′-UTR fragments used in the luciferase assay are indicated as 3′-UTR1 and 3′-UTR2. **b**–**d** Luciferase assay showing that CPEB3 can inhibit the luciferase expression of the reporters containing 3′-UTR1 or 3′-UTR2 (**b**), but not the reporters containing a reverse complementary 3′-UTR (**c**) or lacking the 3′-UTR (**d**). **e** High enrichment of RIP-Seq reads in the 3′-UTR of the MTDH mRNA in IP-FLAG samples compared with IgG control samples. *P < 0.05, **P < 0.01, and ns not significant.

It has been well established that CPEBs controls the translation of its targeted mRNAs^31^. Notably, the in-depth analysis of the 3′-UTR of MTDH mRNA showed multiple nucleotide elements (CPEC, Hex, CPENC, and PBE) distributed in two clusters, which named as 3′-UTR1 (10–2014 bp) and 3′-UTR2 (2444–4358 bp) (Fig. 4a). To address whether CPEB3 inhibited the translation of MTDH mRNA through interacting with these CPE elements, we constructed luciferase reporter vectors containing the proximal (3′-UTR1) or distal (3′-UTR2) region of MTDH mRNA 3′-UTR, as well as vectors containing the reverse complementary sequences. Those reporter vectors were co-transfected with pcDNA3.1(+)CPEB3-FLAG or pcDNA3.1(+) into HEK293T cells, and luciferase activity was detected. The result showed that the firefly luciferase activity was significantly decreased in the reporter containing 3′-UTR1 and 3′-UTR2 (Fig. 4b), but not those containing the reverse complementary 3′-UTRs (Fig. 4c) or control sequence (Fig. 4d) when CPEB3 was overexpressed. In addition, RIP-Seq reads were highly enriched in the 3′-UTR of the MTDH mRNA transcript in the IP-FLAG samples compared with the IgG control samples (Fig. 4e). These results suggested that CPEB3 interacted with 3′-UTR of MTDH mRNA and therefore inhibited the translation of MTDH mRNA.

### CPEB3 inhibits the progression of HCC through down-regulating MTDH

**Fig. 5:**
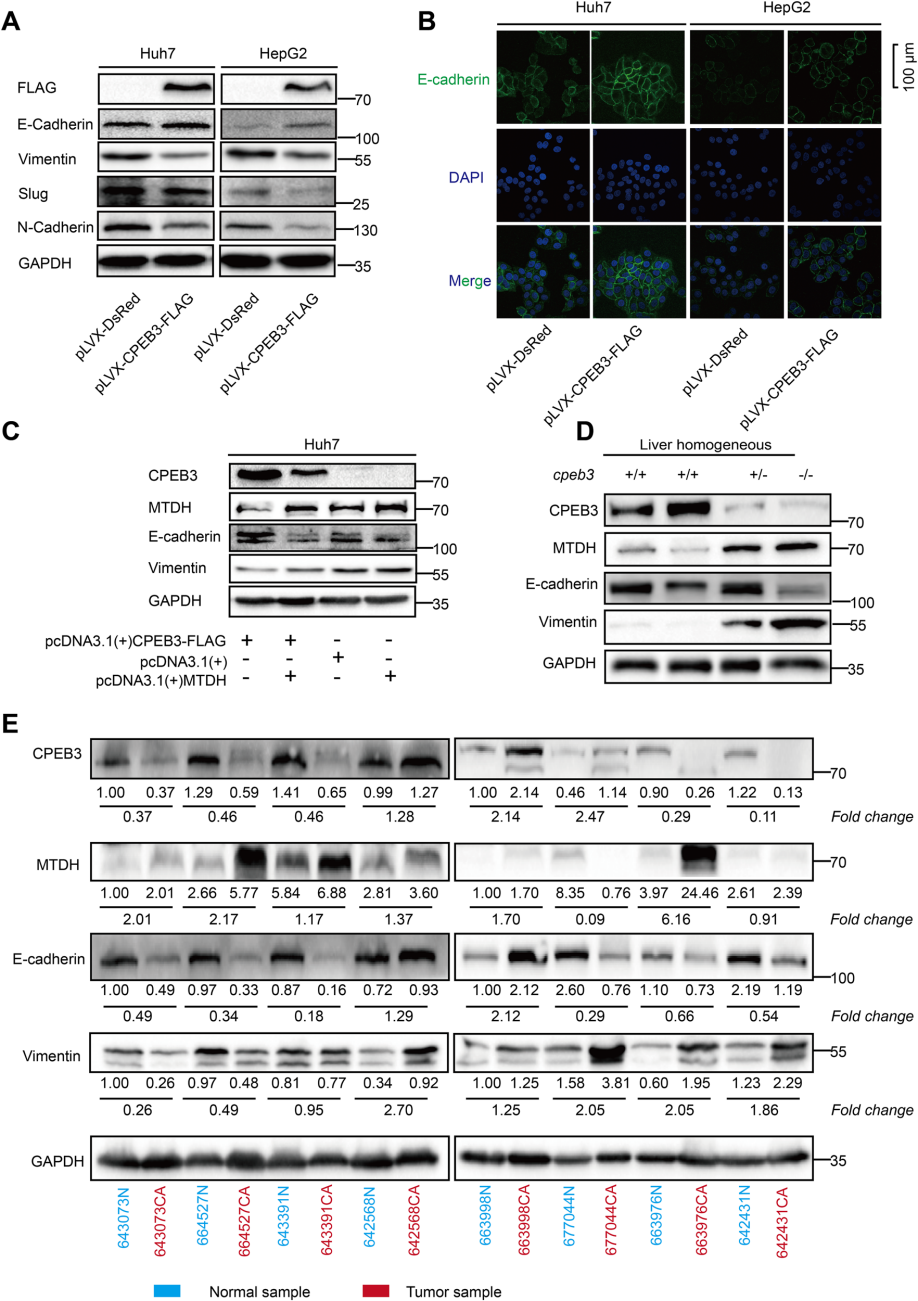
CPEB3 inhibits the progression of HCC through down-regulating MTDH. **a** Western blot analysis of EMT-related markers in CPEB3-overexpression Huh7 and HepG2 cells. **b** Representative immunofluorescence images of E-cadherin expression in CPEB3-overexpressing Huh7 and HepG2 cells. Cell nuclei and E-cadherin are shown in blue and green, respectively. **c** Western blot analysis showing that the forced expression of MTDH could rescue the inhibitory effect of CPEB3 on EMT. **d** Western blot analysis of the EMT-related markers in liver tissue of *cpeb3*^+/+^, *cpeb3*^+/−^, and *cpeb3*^−/−^ mice. **e** The protein expression of CPEB3, MTDH, E-cadherin, and vimentin by Western blot analysis in HCC and paired adjacent normal tissues that collected from the local hospital.

To figure out whether CPEB3-mediated inhibition of HCC cell migration and invasion via suppressing MTDH expression. It has been reported that MTDH promotes metastasis of HCC cells by promoting EMT^18^. First, to verify the assumption that CPEB3 can suppress HCC cell EMT, Western blot and IF were utilized to detect EMT markers expression, including E-cadherin, N-cadherin, vimentin, and Slug (Fig. 5a, b). Next, to determine the contribution of CPEB3-mediated MTDH translational repression to EMT, we performed the “rescue” experiment. The result demonstrated that the HCC cells change back to mesenchymal-like from epithelial-like induced by CPEB3 when MTDH is overexpressed (Fig. 5c). In addition, we also found that the protein expression of E-cadherin and vimentin changed with MTDH protein level in liver tissue of *cpeb3* KO mice (Fig. 5d). We also analyzed the protein expression of CPEB3, MTDH, vimentin, and E-cadherin in eight pairs of human HCCs and matched adjacent normal tissues. We observed that CPEB3 was significantly downregulated in HCC compared with the matched adjacent normal tissue. Notably, CPEB3 expression is inversely proportional to MTDH and vimentin expression while proportional to E-cadherin expression among the tested samples (Fig. 5e). These results suggested that CPEB3 serves as a negative regulator of EMT, thereby promoting HCC toward less metastatic.

**Fig. 6:**
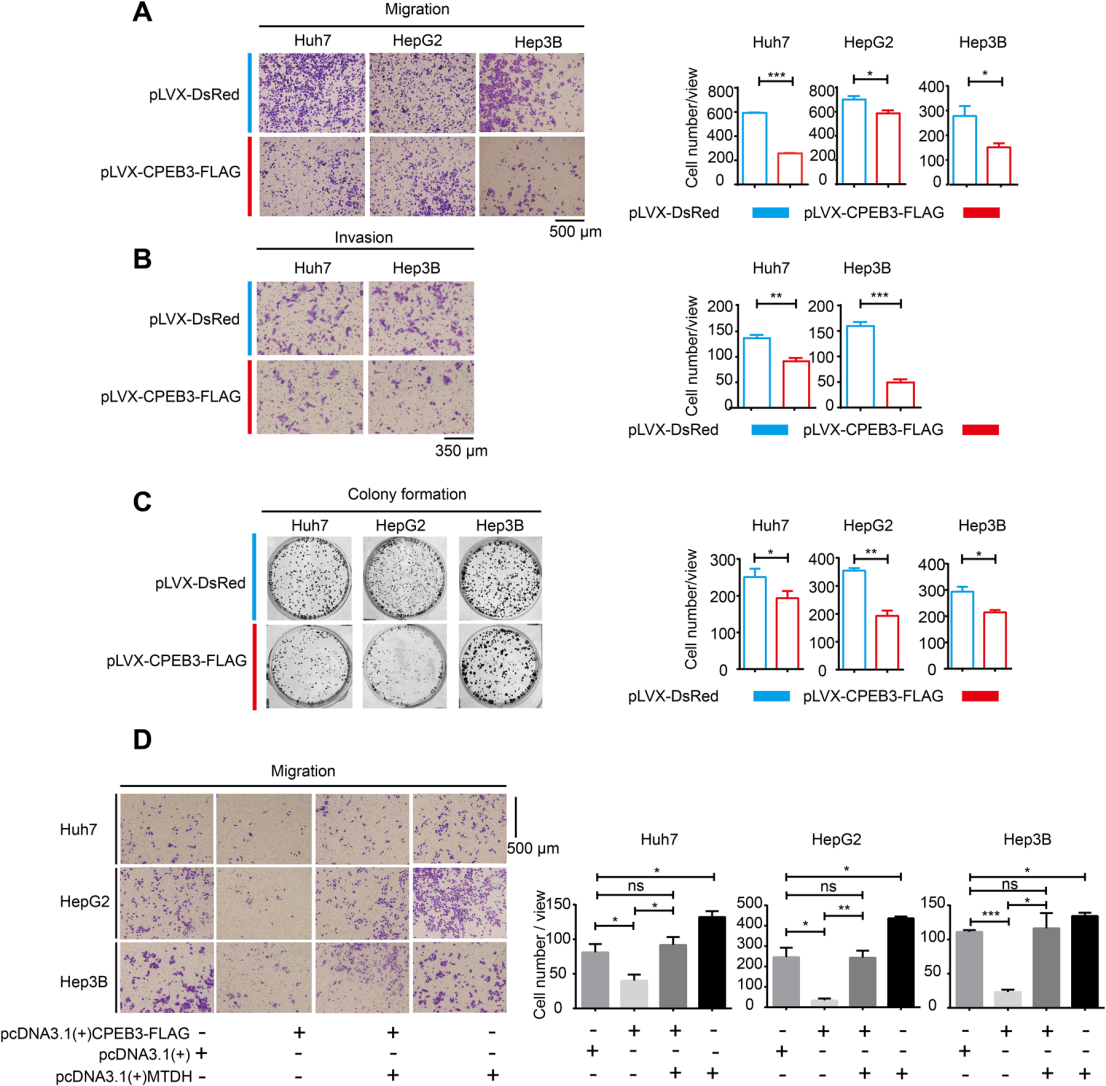
CPEB3 inhibits cell migration, invasion and colony formation in HCC cells. **a** Transwell assay showing that CPEB3 overexpression inhibited the migration of HCC cells (magnification, ×60; independent Student’s *t* test). **b** Transwell assay showing that CPEB3 overexpression inhibited the invasive ability of HCC cells (magnification, ×120; independent Student’s *t* test). **c** Inhibition of the colony formation ability of the Huh7, HepG2, and Hep3B cell lines after CPEB3 overexpression (independent Student’s *t* test). **d** Transwell assay showing the overexpression of MTDH rescue the inhibitory effect of CPEB on migratory ability of HCC (magnification, ×100; independent Student’s *t* test). For all figures, the results are presented as the mean ± SEM of three independent experiments; *P < 0.05, **P < 0.01, ***P < 0.001, and ns not significant.

It is widely believed that EMT can facilitate the invasion and metastasis of the tumor^32^. Therefore, we examined the role of CPEB3 and MTDH in the migration, invasion, and colony formation of HCC cells. Transwell assay revealed that the overexpression of CPEB3 could strongly inhibit the migratory (Fig. 6a) and invasive (Fig. 6b) abilities of HCC cells. Similarly, the migratory ability of Huh7 cells was boosted when CPEB3 expression was stably knocked down (Supplementary Fig. S2A, B). Transwell assay using medium containing mitomycin c also indicated that CPEB3-mediated migration inhibition independently with the CPEB3-induced proliferation change of the cells (Supplementary Fig. S2D). In addition, we found that HCC cells with high CPEB3 expression had relatively low colony formation ability (Fig. 6c). Similarly, colony formation ability was substantially boosted when CPEB3 expression was knocked down (Supplementary Fig. S2C). The “rescue” experiment showed that MTDH could partly reverse CPEB3-mediated migration inhibition of HCC cells (Fig. 6d). These findings indicated that the translational suppression of MTDH mediated by CPEB3 played a role in inhibiting migration and invasion of HCC cells.

### *cpeb3* KO mice are vulnerable to carcinogen-induced hepatocarcinogenesis and subsequent lung metastasis

**Fig. 7:**
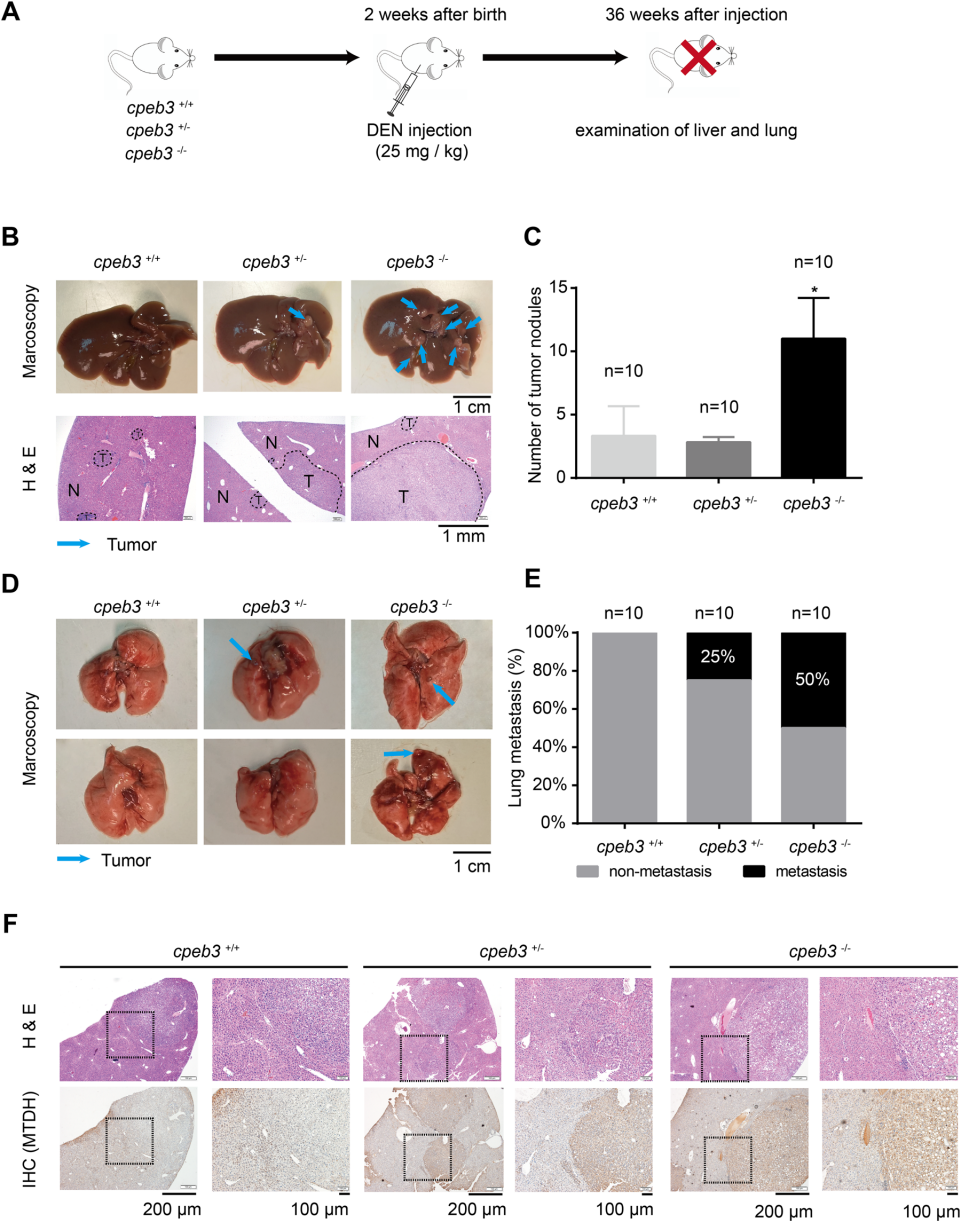
cpeb3 knockout mice are vulnerable to DEN-induced hepatocarcinogenesis and subsequent lung metastasis. **a** The workflow for DEN-induced HCC model. **b** Representative macroscopy images and H&E staining of the fresh livers from *cpeb3*^+/+^, *cpeb3*^+/−^, and *cpeb3*^−/−^ mice 36 weeks after the DEN injection. The tumor foci were marked as blue arrows; T tumor, N non-tumor tissue. **c** The number of tumor nodules (diameter >1 mm) were counted. (the results are presented as the mean ± SEM, Student’s *t* test; *P < 0.05). **d** Representative images of the lung in *cpeb3*^+/+^, *cpeb3*^+/−^, and *cpeb3*^−/−^ mice 36 weeks after the DEN injection. The tumor foci were indicated as blue arrows. **e** The proportion of mice with or without lung metastasis in each genotype were summarized by the bar plot. **f** Immunohistochemistry (IHC) showing MTDH protein expression in cancerous tissue and adjacent normal tissue in *cpeb3*^+/+^*, cpeb3*^+/−^, and *cpeb3*^−/−^ mice.

We adopted a DEN-induced HCC model by a single intraperitoneal injection of DEN at 13 days after birth. The treated mice were sacrificed 36 weeks after injection, then the livers and lungs were dissected and examined (Fig. 7a). We found that *cpeb3*^−/−^ mice have more tumor foci in the liver compared with the *cpeb3*^+/−^ and *cpeb3*^+/+^ mice (Fig. 7b, upper panel). Besides, the size of the tumors in *cpeb3*^−/−^ mice were significantly larger than that of the *cpeb3*^+/−^ and *cpeb3*^+/+^ mice (Fig. 7b, lower panel). More importantly, the examination of the lungs indicated that *cpeb3*^−/−^ mice were more vulnerable to lung metastasis compared with *cpeb3*^+/−^ and *cpeb3*^+/+^ mice (Fig. 7d, e). Furthermore, IHC showed that MTDH protein was elevated in the liver tissue of *cpeb3*^+/−^ and *cpeb3*^−/−^ mice compared with the *cpeb3*^+/+^ mice (Fig. 7f). Notably, in *cpeb3*^+/−^ and *cpeb3*^−/−^ mice, MTDH protein was significantly upregulated in the cancerous tissue compared with the adjacent normal tissue (Fig. 7f). Those results demonstrated that *cpeb3* is a protective gene for hepatocarcinogenesis and the subsequent lung metastasis.

### CPEB3 and its targets are associated with the progression and prognosis of HCC

**Fig. 8:**
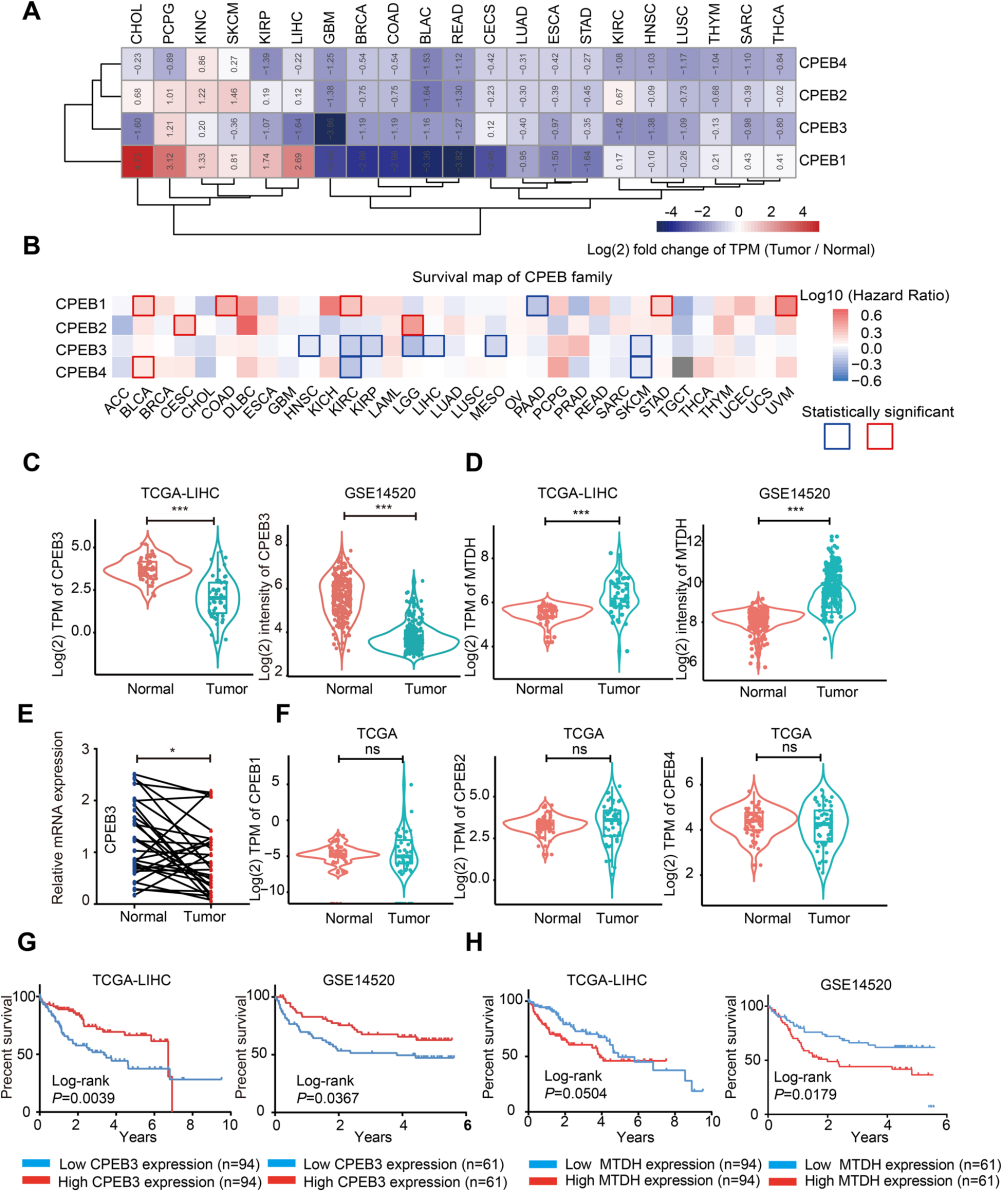
CPEB3 and its targets are associated with the progression and prognosis of HCC. **a** Heat map of the log 2 fold changes (tumor/normal) of the expression of CPEB1–4 across 21 types of malignancies. **b** Survival map showing the correlation between the overall survival time and the expression of the CPEB family of proteins. **c** Violin plot of CPEB3 expression in 50 paired tumor and normal tissue samples in the TCGA-LIHC dataset (paired Student’s *t* test) and GSE14520 dataset (tumor = 247, normal = 239, independent Student’s *t* test). **d** Violin plot showing MTDH mRNA expression in paired (TCGA-LIHC, tumor *n* = 50, normal *n* = 50, paired Student’s *t* test) and unpaired (GSE14520, tumor *n* = 247, normal *n* = 239, independent Student’s *t* test) tumor/normal samples. **e** Expression of CPEB3 by qRT-PCR in 30 paired HCCs and adjacent normal tissue samples (paired Student’s *t* test). The relative expression of CPEB3 mRNA in tumor and adjacent normal tissues were shown as blue and red dots, respectively. **f** Violin plots showing the expression of CPEB1, CPEB2, and CPEB4 in 50 paired tumor and normal tissue samples in the TCGA-LIHC dataset (ns: no significant difference, paired Student’s *t* test). **g** Kaplan–Meier analysis of patients with different CPEB3 expression in the TCGA-LIHC dataset and GSE14520 (log-rank test). **h** Kaplan–Meier analysis of patients with different MTDH expression in the TCGA-LIHC dataset and GSE14520 (log-rank test). For all figures, *P < 0.05, and ***P < 0.001. TCGA The Cancer Genome Atlas, LIHC liver hepatocellular carcinoma; the abbreviations of TCGA studies can be found in (https://gdc.cancer.gov/).

It can be concluded from the above results that CPEB3 can restrain tumor progression by regulating a large number of target genes related to tumor metastasis. To verify the universality of CPEB dysregulation in different types of malignancy, we analyzed the expression pattern of CPEBs in the TCGA database. We found that the dysregulation of CPEBs was a common phenomenon across most malignancies (Fig. 8a). Importantly, CPEB3 was downregulated in 18 out of 21 analyzed malignancies, including HCC (Fig. 8a). In addition, survival analysis showed that CPEB3 was a protective molecule in 7 out of 33 analyzed malignancies and it was the only prognosis-related molecule in the CPEB family in HCC (Fig. 8b). We then examined the CPEB3 and MTDH expression in two independent cohorts of samples, TCGA-LIHC and GSE14520. CPEB3 was significantly downregulated (Fig. 8c) and MTDH was significantly upregulated (Fig. 8d) in HCC compared with normal samples. Consistent with the in silico data, an analysis of fresh samples collected from the local hospital also showed that CPEB3 expression was significantly downregulated in HCC compared with adjacent normal tissues (Fig. 8e). However, the expression analysis of CPEB1, CPEB2, and CPEB4 in 50 HCCs and matched normal samples did not display significant difference (Fig. 8f), which may imply that CPEB3 and its targets exerted special functions during HCC progression. Consistent with the above results, the patients with high CPEB3 expression have a more optimistic overall survival time in two independent datasets, TCGA-LIHC and GSE14520 (Fig. 8g). The high level of MTDH expression was linked with worse prognosis in HCC patients (Fig. 8h). Collectively, these data suggested that the regulation relationship between CPEB3 and its target, MTDH, is associated with the progression, aggressiveness, and prognosis of HCC.

## Discussion

HCC is a life-threatening disease. Most importantly, the recurrence and metastasis of HCC are the major causes of death. Although HCC progression has been associated with several genetic and epigenetic alterations, it is now generally believed that post-transcriptional regulation is also very important in the regulation of malignant transformation^33^. Cytoplasmic polyadenylation is a mechanism to affect mRNA translation, which is driven by CPEBs. CPEBs can modulate translational activation or repression by binding to CPE mainly found in 3′-UTR. Several studies demonstrate that CPEB family members mediate malignant transformation, including glioma, colorectal cancer, and so forth^34^. While our research group firstly reported that CPEB3 involved in the regulation of liver cancer progression^12^. Herein, we showed that CPEB3 interacted with hundreds of mRNAs to regulate their translation through post-transcriptional modification in HCC cells (Supplementary Table S6). Impressively, ORA enrichment analyses showed that CPEB3 binders in HCC were enriched in cancer-related pathways, including hippo signaling, ERK signaling, TGF-β signaling, FoxO signaling, and cell senescence. Considering that 20% of vertebrate transcriptomes are under the control of cytoplasmic polyadenylation^35,36^, the dysregulation of CPEB3 may alter the translation of a broad range of proteins resulting in the formation of a tumor-promoting environment that contributes to the progression of HCC. It is worth noting that many metastasis-related mRNAs are targeted by CPEB3, which may contribute to the anti-metastatic function of CPEB3. Actually, we did identify a transcript that enriched in the CPEB3 immunoprecipitant, MTDH, which is regulated by CPEB3. More importantly, CPEB3 can repress the EMT and metastasis of HCC cells by repressing the translation of MTDH mRNA.

CPEBs are important in the control of poly(A) tail elongation or removal, which belongs to post-transcriptional modification^31^. Although CPEB1–4 share highly conserved motifs in the C terminus, which are crucial for their RNA recognition and binding, CPEBs are believed to work differently in a tissue-dependent manner^8,35^. By conducting RIP-Seq, researchers have found that the binding spectrum of CPEB4 is highly lineage dependent, as there is little overlap among CPEB4-bound mRNAs between melanoma and pancreatic cancer^8,37^. It is well known that CPEB3 targets multiple transcripts involved in regulating neural-related functions^4,10,38–41^. However, enrichment analysis of our data showed that the CPEB3 binders in HCC were less involved in the neural system. This finding suggested that the binding spectrum of CPEB3 was also highly variable across different tissues, which indicated that CPEB3 may have a distinct functional role in HCC compared with the nervous system. Indeed, our experiments demonstrated that the function of CPEB3 in HCC was evident, as CPEB3-overexpressed HCC cells exhibited a reduced metastatic ability compared with the control group.

MTDH, also known as AGE1 and LYRIC, is a multifunctional protein that was first discovered in astrocytes with a high expression level^14^. Intensive efforts have been made to reveal the essential function of MTDH in promoting tumor progression. MTDH is frequently found to have elevated expression in high-grade and/or metastatic tumors^14^. The overexpression of MTDH leads HCC cells to become more aggressive and metastatic^17^. However, the underlying mechanisms leading to the high level of MTDH in metastatic HCC have not been established. Similar to the previous reports, our data suggested that MTDH mRNA was upregulated in HCCs and negatively linked to prognosis. We proposed that the downregulation of CPEB3 expression may partially contribute to the high level of MTDH protein in scenarios such as metastasis in HCC. We observed that the migratory abilities were restored when MTDH was reintroduced into CPEB3-overexpressing HCC cell lines. Although other metastasis-related mRNAs may also be targeted by CPEB3, MTDH seems to play a more dominant role in mediating the anti-migratory function of CPEB3 in HCC cells.

Interestingly, we found that the protein level of MTDH was also upregulated in *cpeb3*-null mice instead of the mRNA level. Besides, we also observed low expression of E-cadherin and high expression of vimentin in the liver tissue of *cpeb3*^−/−^ mice compared with that of *cpeb3*^+/+^ mice. This indicated that CPEB3-mediated translational suppression of MTDH is not restricted in malignant HCC cells and can be at least adopted in normal hepatocytes. Most importantly, we found that *cpeb3*^−^^/−^ mice are more susceptible to DEN-induced hepatocarcinogenesis. The more lung metastasis foci were also found in the *cpeb3*^−/−^ mice than *cpeb3*^+/+^ mice. Further, MTDH protein expression was significantly elevated in the cancerous tissue compared with the adjacent normal tissue in the liver of *cpeb3*^−/−^ and *cpeb3*^+/^^−^ mice, but not the *cpeb3*^+/+^ mice. We speculate that CPEB3 suppressed the translation of MTDH mRNA in both normal and cancerous tissue in *cpeb3*^+/+^ mice; however, when this translational suppression was relieved in the *cpeb3*^+/−^ or *cpeb3*^−/−^ mice, the elevation of MTDH protein in the cancerous tissue became more prominent. This in vivo observation further supported the notion that CPEB3 inhibited hepatocarcinogenesis as well as tumor metastasis.

In summary, identifying the role of CPEB3 in HCC provides us with a novel molecular index for the diagnosis and prognosis of HCC, which makes CPEB3 possible to be involved in therapeutic applications. While one limitation of our study is that the change of MTDH mRNA poly(A) tail length mediated by CPEB3 overexpression has not been described, which needs to be characterized in the future research. Our data suggested that loss of CPEB3 is one of the reasons why HCC cells acquire metastatic potential. Taking advantage of RIP and high-throughput sequencing, we revealed the binding spectrum of CPEB3 in HCC for the first time. The RIP-Seq data may provide valuable information for future work on elucidating the anticancer role of CPEB3. Our work also extends the boundary of the understanding of post-transcriptional regulation, as we illustrate the pivotal impact of CPEB3 on HCC metastasis.

## Supplementary information

Supplementary Figure Legends

Supplementary Materials and Methods

Figure S1

Figure S2

Supplementary Table S2

Supplementary Table S3

Supplementary Table S4

Supplementary Table S5

Supplementary Table S1

Supplementary Table S6

Supplementary Table S7

## Data Availability

The RIP-Seq RAW data in this study is available from the corresponding author upon reasonable request.

## Code availability

The code for bioinformatics analysis in this study is available from the corresponding author upon reasonable request.
