# Supplementary material for: CPEB3-mediated MTDH mRNA translational suppression restrains hepatocellular carcinoma progression
Source: Cell Death Dis. 2020 Sep 23;11(9):792. doi: 10.1038/s41419-020-02984-y (PMC7511356; doi:10.1038/s41419-020-02984-y)
Supplement: Supplementary file 1 — Supplementary Figure Legends [file 41419_2020_2984_MOESM1_ESM.doc]

**Figure S1. Construction and verification of *cpeb3* knockout mice**

**(A)** The design of mouse *cpeb3* specific sgRNA. The sgRNA was designed to target the second exon of the mouse *cpeb3* gene, the targeting site resides in the 5’ proximal region of CPEB3 open reading frame to achieve the higher knockdown efficiency. **(B)** Verification of gene editing in F0 funders by T7E1 assay. The cleaved bands indicating the succeed introducing of mutations at sgRNA targeted site, as marked by red arrow. **(C)** Genotyping of *cpeb3*^+/+^, *cpeb3*^+/-^ and *cpeb3*^-/-^ mice by DNA sequencing.

**Figure S2. Knockdown of CPEB3 promotes migration and colony formation of HCC cells**

**(A)** Verification of stable CPEB3 knockdown cell lines by western blot and qRT-PCR (independent Student’s *t-test*). **(B)** CPEB3 knockdown promoted migration of Huh7 cells (magnification, 60×; independent Student’s *t-test*). **(C)** CPEB3 knockdown promoted colony formation of Huh7 cells (independent Student’s *t-test*). **(D)** CPEB3 inhibits the migration of Huh7 and HepG2 cells independently with cell proliferation (magnification, 60×; independent Student’s *t-test*). For all figures, the results are summarized as the mean ± SEM of three independent experiments; * *P* < 0·05, ** *P* < 0·01 and ****P* < 0.001.
