# Supplementary material for: CPEB3-mediated MTDH mRNA translational suppression restrains hepatocellular carcinoma progression
Source: Cell Death Dis. 2020 Sep 23;11(9):792. doi: 10.1038/s41419-020-02984-y (PMC7511356; doi:10.1038/s41419-020-02984-y)
Supplement: Supplementary file 2 — Supplementary Materials and Methods [file 41419_2020_2984_MOESM2_ESM.doc]

**Supplementary** **materials and methods**

**1.1 The criteria for data exclusion in TCGA and GEO data analysis.**

The GSE9843 dataset (Gene expression profiling of 91 hepatocellular carcinomas with hepatitis C virus etiology) contains 91 HCC samples, according to the clinical information in the Series Matrix, 45 samples classified as “vascular invasion--- Yes”, 34 samples classified as “vascular invasion---NO”, 12 samples classified as “vascular invasion--NA”, we select the 79 samples for analysis.

The GSE14520 dataset (Gene expression data of human hepatocellular carcinoma) contains 488 samples, according to the clinical information in the “Extra-supplements.txt” in the GSE dataset, 239 samples classified as “Non-Tumor”, 247 samples classified as “Tumor”, these samples were involved in the analysis. There were 2 samples classified as “Normal”, which were excluded from the analysis.

The GSE20017 dataset (Gene Signature to Identify Vascular Invasion in Hepatocellular Carcinoma) contains 135 samples, according to the clinical information in the Series Matrix, 40 samples classified as “vascular invasion --- Yes”, 95 samples classified as “vascular invasion --- No”, all the samples were subjected to the analysis.

The TCGA-LIHC dataset contains 371 samples. For mRNA expression analysis in the paired Tumor/Normal samples, we selected them according to the TCGA barcode definition (https://docs.gdc.cancer.gov/Encyclopedia/pages/TCGA_Barcode), a total 50 paired normal and tumor samples were selected and subjected to the analysis.
