# Supplementary figures and images for: CPEB3-mediated MTDH mRNA translational suppression restrains hepatocellular carcinoma progression
Source: Cell Death Dis. 2020 Sep 23;11(9):792. doi: 10.1038/s41419-020-02984-y (PMC7511356; doi:10.1038/s41419-020-02984-y)

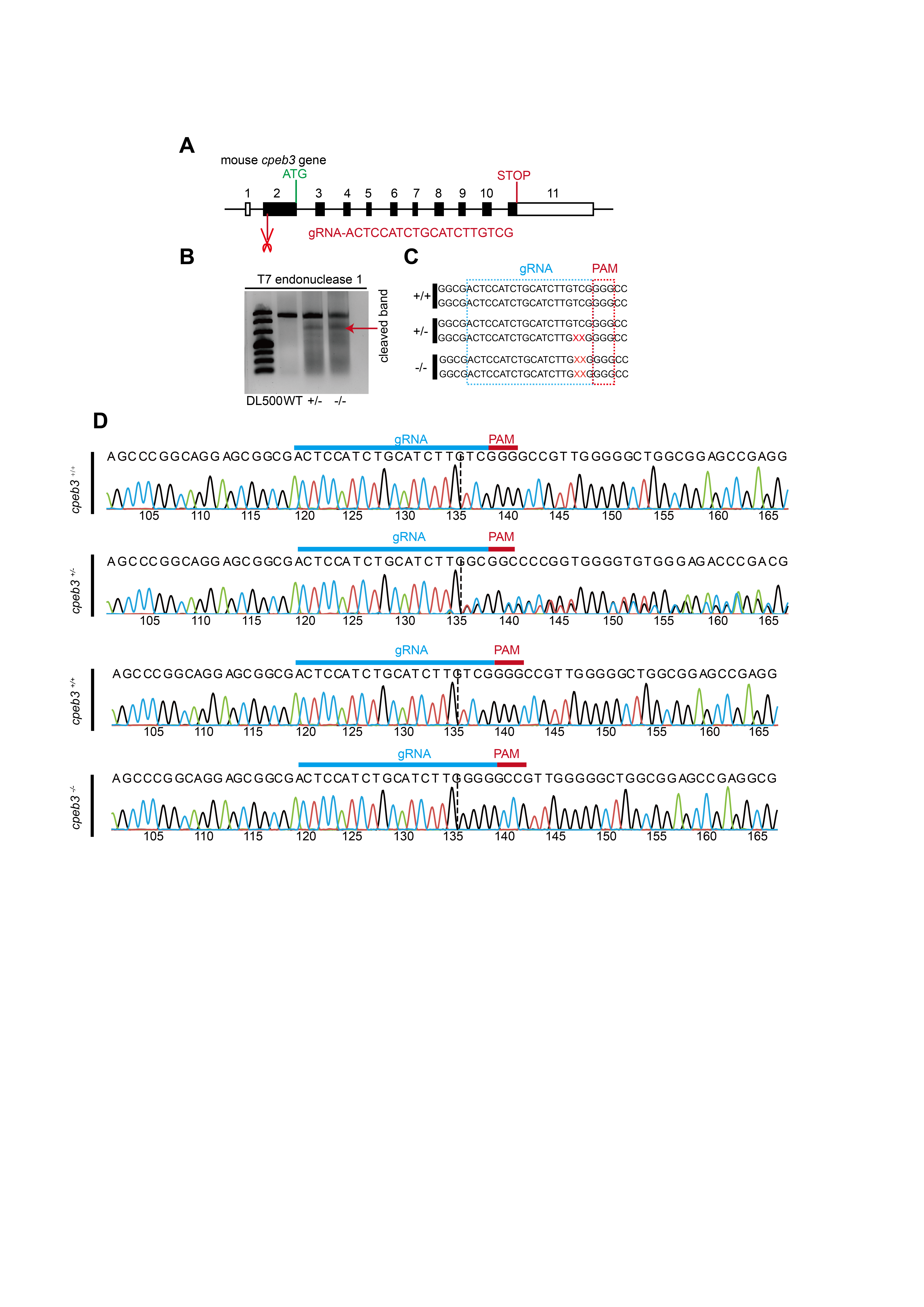

Supplement: Supplementary file 3 — Figure S1 [file 41419_2020_2984_MOESM3_ESM.tif]

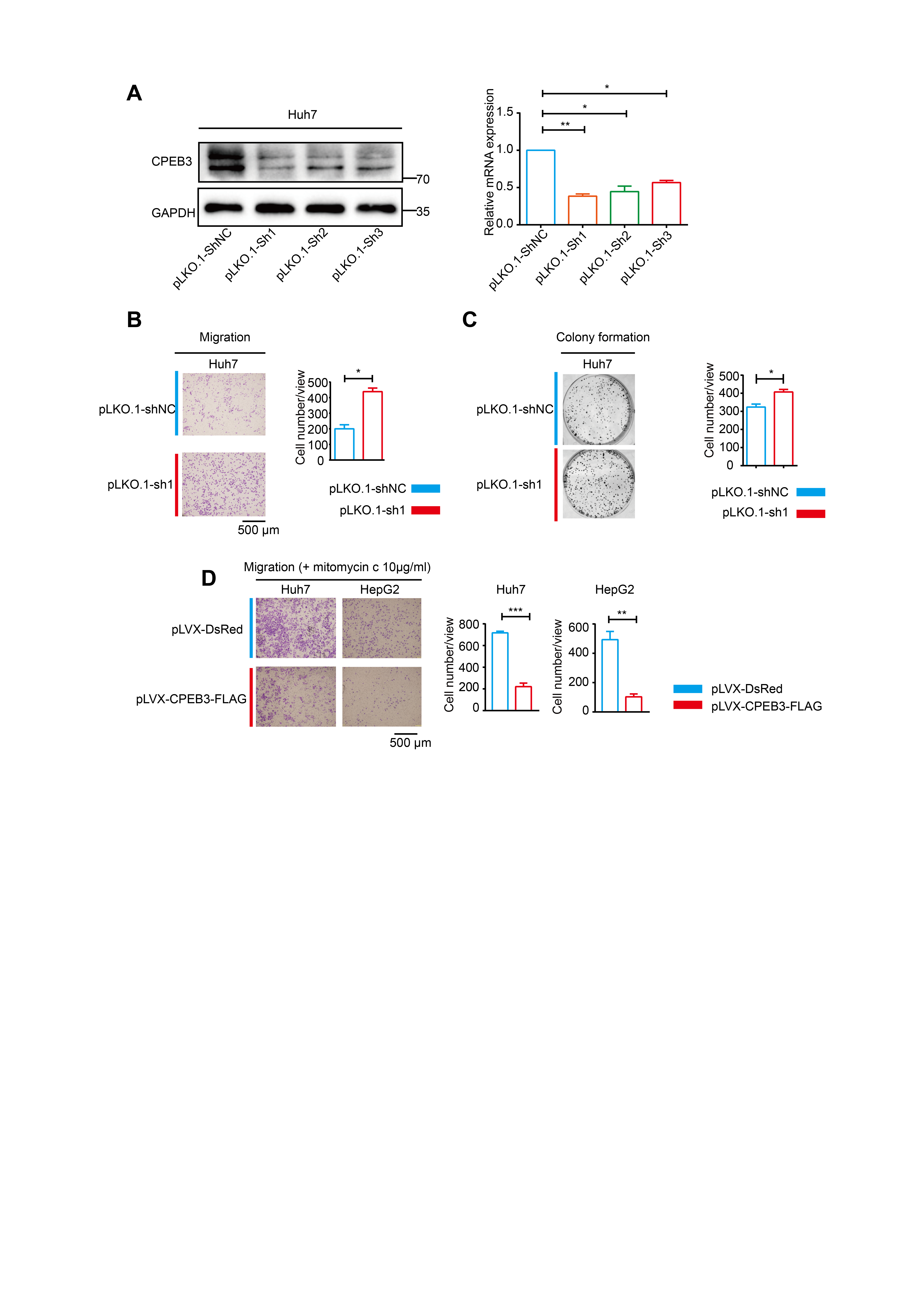

Supplement: Supplementary file 4 — Figure S2 [file 41419_2020_2984_MOESM4_ESM.tif]
