# Supplementary material for: CPEB3-mediated MTDH mRNA translational suppression restrains hepatocellular carcinoma progression
Source: Cell Death Dis. 2020 Sep 23;11(9):792. doi: 10.1038/s41419-020-02984-y (PMC7511356; doi:10.1038/s41419-020-02984-y)
Supplement: Supplementary file 5 — Supplementary Table S2 [file 41419_2020_2984_MOESM5_ESM.docx]

**Supplementary Table S2. Oligonucleotides used in this study**

| Name | Sequence (5’-3’) |  |
| --- | --- | --- |
| pLKO.1-sh1 | F:CCGGAAGTTCTGGAAGAGCATGGTTCTCGAGAACCATGCTCTTCCA  GAACTTTTTTTG |  |
|  | R:AATTCAAAAAAAGTTCTGGAAGAGCATGGTTCTCGAGAACCATGCTCTTCCAGAACTT |  |
| pLKO.1-sh2 | F:CCGGGAGGATAACGCTTTCCGGACCGATACTCGAGTATCGGTCCGGAAAGCGTTATCCTCTTTTTG |  |
|  | R:AATTCAAAAAGAGGATAACGCTTTCCGGACCGATACTCGAGTATCGGTCCGGAAAGCGTTATCCTC |  |
| pLKO.1-sh3 | F:CCGGGGTGTCTGCTATGCTGGCATTGATACTCGAGTATCAATGCCAGCATAGCAGACACCTTTTTG |  |
|  | R:AATTCAAAAAGGTGTCTGCTATGCTGGCATTGATACTCGAGTATCAATGCCAGCATAGCAGACACC |  |
| pLKO.1-shNC | F:CCGGCCGCAGGTATGCACGCGTCTCGAGACGCGTGCATACCTGCGGTTTTTTG |  |
|  | R:AATTCAAAAAACCGCAGGTATGCACGCGTCTCGAGACGCGTGCATACCTGCGG |  |
