# Supplementary material for: CPEB3-mediated MTDH mRNA translational suppression restrains hepatocellular carcinoma progression
Source: Cell Death Dis. 2020 Sep 23;11(9):792. doi: 10.1038/s41419-020-02984-y (PMC7511356; doi:10.1038/s41419-020-02984-y)
Supplement: Supplementary file 6 — Supplementary Table S3 [file 41419_2020_2984_MOESM6_ESM.docx]

**Supplementary Table S3. Primers used in this study**

| Name | Sequence (5’-3’) |
| --- | --- |
| CPEB3 | F: CAGCCTCCTCAGCAGCC |
|  | R: CCCTGGAAGAAGCTGTCCTC |
| CPEB1 | F: CCTGGGTATTAGCCGACAGT |
|  | R: GCCTCAGCATTTAGCATTCC |
| CPEB2 | F: TGCTATGATCATGGACCGGC |
|  | R: TGGCTTTACCTCCACACGTT |
| CPEB4 | F: TGGGGATCAGCCTCTTCATA |
|  | R: CAATCCGCCTACAAACACCT |
| MCL1 | F: TTCCAGTAAGGAGTCGGGGT |
|  | R: CCTCCTTCTCCGTAGCCAAAA |
| CDK1 | F: GCGGAATAATAAGCCGGGATCT |
|  | R: AGGAACCCCTTCCTCTTCACT |
| MTDH | F: AAATAGCCAGCCTATCAAGACTC |
|  | R: TTCAGACTTGGTCTGTGAAGGAG |
| TOMM20 | F: GCCCTTAGTTGACTGGGACC |
|  | R: TCCGACCCACCATCTTCTCT |
| FOXQ1 | F: CGCGGACTTTGCACTTTGAA |
|  | R: AGCTTTAAGGCACGTTTGATGGAG |
| ID3 | F: CTGCTACGAGGCGGTGTG |
|  | R: CACCTGGCTAAGCTGAGTGC |
| ACTIN | F: TACCTCATGAAGATCCTCACC |
|  | R: TTTCGTGGATGCCACAGGAC |
| CTNNB1 | F: GCTGGGACCTTGCATAACCTT |
|  | R: ATTTTCACCAGGGCAGGAATG |
| GAPDH | F: CGCTCTCTGCTCCTCCTGTT |
|  | R: CCATGGTGTCTGAGCGATGT |
| MTDH-Clone | F:CGGAATTCCGTTCCTCGCTTCCCTCGACTA |
|  | R:GCTCTAGAGCTATGGTGGTGTCCGCAGTTT |
| MTDH-UTR1 | F: ATAGAGCTCCCTGAATTGGACATGTGTTTG |
|  | R: GCTCTAGAGCCATCTCTGCATCTCCACAAT |
| MTDH-UTR2 | F: ATAGAGCTCTCTTTCACATGCATGAGGTG |
|  | R: GCTCTAGAGCCTGAGGCTAAAGTGGGAGAATA |
| MTDH-RUTR1 | F: GCTCTAGAGCCCTGAATTGGACATGTGTTTG |
|  | R: ATAGAGCTCCATCTCTGCATCTCCACAAT |
| MTDH-RUTR2 | F: GCTCTAGAGTCTCTTTCACATGCATGAGGTG |
|  | R: ATAGAGCTCCTGAGGCTAAAGTGGGAGAATA |
