# Supplementary material for: CPEB3-mediated MTDH mRNA translational suppression restrains hepatocellular carcinoma progression
Source: Cell Death Dis. 2020 Sep 23;11(9):792. doi: 10.1038/s41419-020-02984-y (PMC7511356; doi:10.1038/s41419-020-02984-y)
Supplement: Supplementary file 7 — Supplementary Table S4 [file 41419_2020_2984_MOESM7_ESM.docx]

**Supplemental Table S4. Antibodies used in this study**

| Target | Species | Dilution (Application) | Manufacture |
| --- | --- | --- | --- |
| CPEB3 | Rabbit | 1: 1000 (WB) | Thermo Fisher Scientific, Waltham, MA, USA  (PA5-44273) |
| E-cadherin | Rabbit | 1: 500 (WB)  1: 100 (IF) | CST, Danvers, MA, USA (#3195) |
| FLAG | Mouse | 1: 2000 (WB) | GenScript, Piscataway, NJ, USA (A00187) |
| GAPDH | Mouse | 1: 1000 (WB) | Thermo Fisher Scientific, Waltham, MA, USA (AM4300) |
| MTDH | Rabbit | 1: 1000 (WB)  1 :200 (IHC) | Abcam, Cambridge, MA, USA (ab45338) |
| N-cadherin | Rabbit | 1: 500 (WB) | CST, Danvers, MA, USA (#13116S) |
| SLUG | Rabbit | 1: 500 (WB) | CST, Danvers, MA, USA (#9585S) |
| Vimentin | Rabbit | 1: 1000 (WB)  1 :100 (IHC) | CST, Danvers, MA, USA (#5741S) |
| IgG (HRP) | Goat | 1: 4000 (WB) | Thermo Fisher Scientific, Waltham, MA, USA  (A24464, A24524) |
| IgG (Alexa 488) | Goat | 1: 100 (IF) | Zhongshan Golden Bridge Bio-technology, China (#ZF0511) |
