# Supplementary material for: CPEB3-mediated MTDH mRNA translational suppression restrains hepatocellular carcinoma progression
Source: Cell Death Dis. 2020 Sep 23;11(9):792. doi: 10.1038/s41419-020-02984-y (PMC7511356; doi:10.1038/s41419-020-02984-y)
Supplement: Supplementary file 8 — Supplementary Table S5 [file 41419_2020_2984_MOESM8_ESM.docx]

**Supplementary Table S5. RNA-Seq preprocessing information**

| Sample name | Raw reads | Ribosomal RNA | Mapped reads | Unique mapped reads |
| --- | --- | --- | --- | --- |
| IP-FLAG1 | 82984513 | 68178080 (82.16%) | 10815311 | 5994891 |
| IP-FLAG2 | 90978235 | 77140874 (84.79%) | 10779728 | 5695439 |
| Input | 93312828 | 84191060 (90.22%) | 5497847 | 2183219 |
